# Risk factors for early wheezing in preterm infants: a retrospective cohort study

**DOI:** 10.3389/fped.2025.1555834

**Published:** 2025-06-24

**Authors:** Xun Chen, Minjing Yang, Jun Xie, Shengzhu Huang, Xiaolian Qin, Zhijian Pan, Maoling Zhu, Dingyuan Zeng, Yifeng Huang, Tao Liang, Chunmei Liang, Guangmin Nong

**Affiliations:** ^1^ Department of Pediatrics, The First Affiliated Hospital of Guangxi Medical University, Nanning, Guangxi, China; ^2^ Department of Student Affairs, Yulin Campus of Guangxi Medical University Yulin, Guangxi, China; ^3^ Center for Genomic and Personalized Medicine, Guangxi Key Laboratory for Genomic and Personalized Medicine, Guangxi Collaborative Innovation Center for Genomic and Personalized Medicine, Guangxi Medical University, Nanning, Guangxi, China; ^4^ Department of Medical Services Section, Maternal & Child Health Hospital of Yulin, Yulin, Guangxi, China; ^5^ Department of Gynecology and Obstetrics, Maternal & Child Health Hospital of Qinzhou, Qinzhou, Guangxi, China; ^6^ Department of Obstetrics, Maternal & Child Health Hospital of Nanning, Nanning, Guangxi, China; ^7^ Department of Gynecology and Obstetrics, Maternal & Child Health Hospital of Liuzhou, Liuzhou, Guangxi, China; ^8^ Department of Gynecology and Obstetrics, Maternal & Child Health Hospital of Guigang, Guigang, Guangxi, China; ^9^ Department of Pediatrics, Maternal & Child Health Hospital of Wuzhou, Wuzhou, Guangxi, China; ^10^ Department of Gynecology and Obstetrics, Maternal & Child Health Hospital of Yuzhou, Yulin, Guangxi, China

**Keywords:** wheezing, preterm infants, risk factors, gestational age, allergy

## Abstract

**Introduction:**

The related factors that cause recurrent wheezing in children are complex, and premature delivery may be one of the reasons. Little is known about early wheezing in preterm infants.

**Methods:**

Data sourced from 1,616 children born between 2007 and 2013 from 8 hospitals of Guangxi in China. All children were followed by telephone or questionnaire through the sixth year of life. Children were grouped by characters of age: Group A: gestational age (GA ≤ 32 weeks, Group B: 32 weeks < GA < 37 weeks, Group C: 37 weeks ≤ GA < 42 weeks.

**Results:**

The incidence and the risk factors of early wheezing in preterm infants were analyzed. The incidence of early wheezing: Group A > Group B > Group C. In Group A, the proportion of small-for-gestational-age (SGA) infant was higher in early wheezing group than in normal group (*P* < 0.05). Male (*95% CI*: 1.611–4.601) and family history of allergy (*95% CI*: 1.222–3.411) were the risk factors for early wheezing in Group B.

**Conclusions:**

Lower gestational age is associated with higher wheezing risk. Preterm infants have higher persistent wheezing incidence than full-term infants. Preterm infants with gestational age <32 weeks have higher transient wheezing incidence than those with gestational age 32–37 weeks or full-term infants. In preterm infants <32 weeks, small for gestational age (SGA) is a potential factor for wheezing. In preterm infants aged 32–37 weeks, male sex, personal allergy history, and family allergy history are potential factors for wheezing, with male sex and family allergy history being significant risk factors.

## 1 Introduction

Asthma is the most common chronic respiratory disease in children. Wheezing is the most typical clinical manifestation of asthma in children. About 57% of children had at least one attack of wheezing at the age of three years (1). Recurring wheezing or asthma affects the growth of children, increases medical costs, and also imposes a larger burden on the family and society (2). According to the progress of the disease, children's wheezing is divided into two types: early wheezing (transient early wheezing and persistent early wheezing) and delayed wheezing (Asthma) (3–5). The related factors that cause recurrent wheezing and even asthma in children are complex, and premature delivery may be one of the reasons. The researches showed that the incidences of wheezing and abnormal lung function were greater in preterm infants than those in full-term infants and premature caused lung damage, lasted for a long time (6). In addition, preterm infants may be related to small airway disease and chronic obstructive pulmonary disease (6–10). Therefore, it is extremely important to identify and prevent early wheezing in preterm infants. This study used stratified analysis and comparison with full-term infants to find out the risk factors that caused early wheezing in preterm infants, with the aim to provide some guidance and help for early identification and effective avoidance of potential risk factors.

## 2 Materials and methods

### 2.1 The sample population

A retrospective cohort study examined 1,616 children (premature and full-term infants) born between 2007 and 2016 from 8 provincial and municipal hospitals in Guangxi of China. All children were followed through the sixth year of life. Children were grouped by characters of age: Group A: gestational age (GA) ≤ 32 weeks, Group B: 32 weeks < GA < 37 weeks, Group C: 37 weeks ≤ GA < 42 weeks.

### 2.2 Data collection

**Figure 1:**
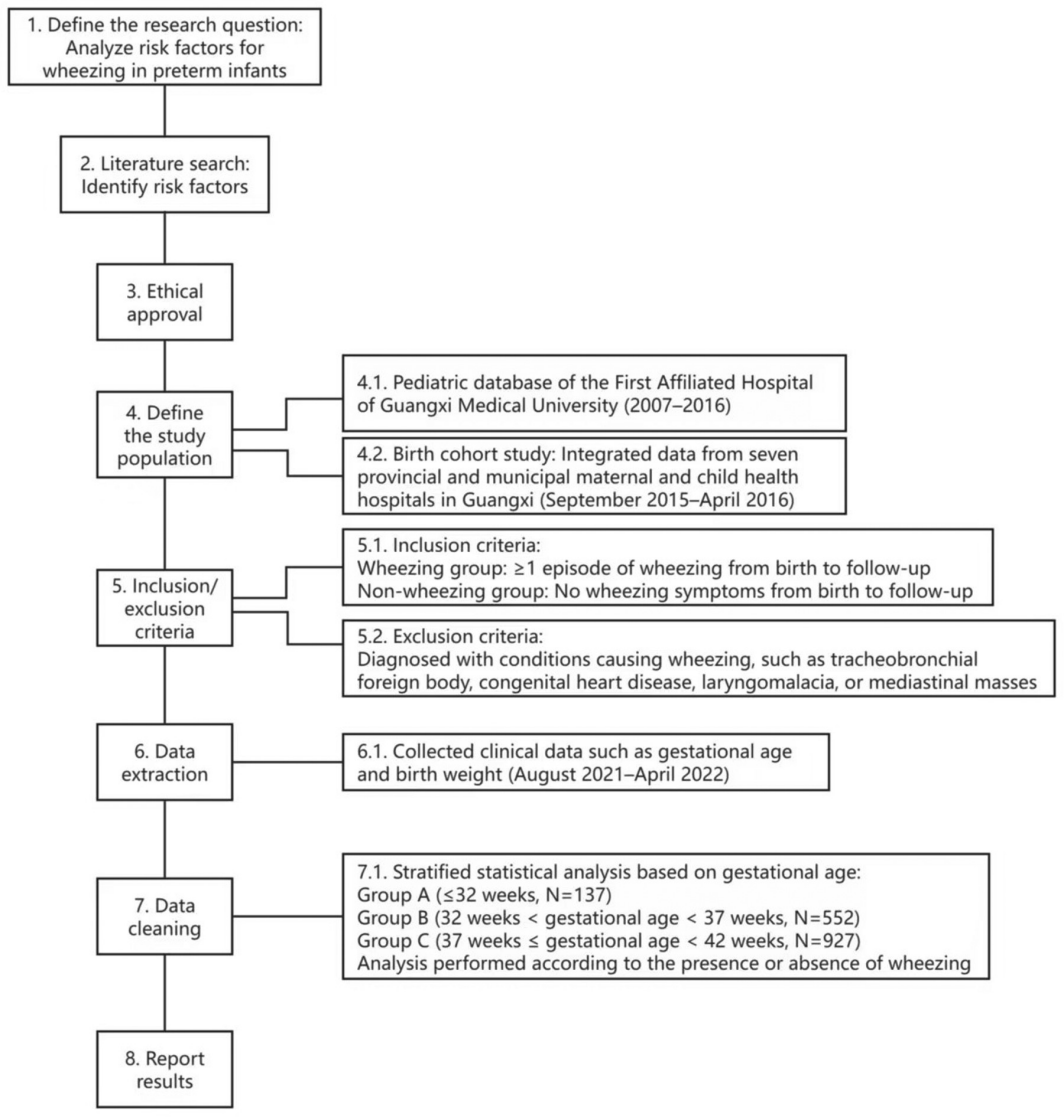
Study flowchart.

The clinical data were collected by telephone or questionnaire (Supplementary Appendix 1). Early wheezing is characterized by the onset of wheezing symptom at or before the age of 3. Transient early wheezing is a type of early wheezing, which gradually disappears before the age of 3, while persistent early wheezing continues to the age of 6. These diseases that cause early wheezing include bronchiolitis, asthmatic bronchitis, asthmatic bronchopneumonia, bronchopulmonary dysplasia, and excluding tracheobronchial foreign bodies and other congenital diseases, such as congenital heart disease, tracheoesophageal fistula, laryngeal chondroplasia and mediastinal space occupying, tracheobronchial malacia, tracheobronchial stenosis, and vascular ring anomalies through medical history inquiry, imaging examinations, and bronchoscopy. The incidences of early wheezing, transient early wheezing, and persistent early wheezing in each group were compared, and the possible factors for early wheezing in preterm infants were analyzed by stratified analysis. These possible factors include gender, mode of delivery, birth weight (BW), relationship between BW and GA, breast feeding, personal history of allergy, family history of allergy, invasive mechanical ventilation and passive smoking. This was the study flowchart (Figure 1).

### 2.3 Data analysis

Enumeration data was expressed by the number of cases or incidence. Each variable was assigned a value: dichotomous variables were used (0 = no, 1 = yes), and unordered multi-categorical variables were assigned by definition. SPSS version 23.0 used to analyze the data of each group by analysis of variance, chi-square test, *Bonferroni* method for multiple comparisons, univariate analysis and multivariate analysis.

## 3 Results

### 3.1 Comparison of the incidence of early wheezing in each group

The incidence rates of wheezing in Groups A, B, and C were 27.0%, 16.7%, and 6.5% respectively, with statistically significant differences between groups (all *P* < 0.0167) (Table 1, Supplementary Appendix 2).

**Table 1:** Summary of wheezing in each group.

Groups	Early wheezing	No wheezing events	Total	Incidence of early wheezing (%)
Group A	37	100	137	27.0
Group B	92	460	552	16.7
Group C	60	867	927	6.5
Total	189	1,427	1,616	11.7

Grouped by characters of age. Group A: gestational age (GA): ≤32 weeks, Group B: 32 weeks < GA < 37 weeks, Group C: 37 weeks < GA < 42 weeks.

### 3.2 The incidences of persistent and transient early wheezing in each group

The incidence of early persistent wheezing was 13.1% in Group A, 11.2% in Group B, and 1.1% in Group C, while early transient wheezing occurred in 13.8%, 5.4%, and 5.4% of Groups A, B, and C respectively (Table 2). The incidence of early persistent wheezing in group A (*P* < 0.0167) and group B (*P* < 0.0167) was significantly greater than that in group C; and the incidence of early transient wheezing in group A was significantly higher than that in group B (*P* < 0.0167) and group C (*P* < 0.0167) by pairwise comparison (Supplementary Appendix 3, 4).

**Table 2:** Comparison of the incidence of persistent early wheezing and transient early wheezing among each group.

Groups	Persistent early wheezing		Transient early wheezing		Total
	number	%	number	%	
Group A	18	13.1	19	13.8	137
Group B	62	11.2	30	5.4	552
Group C	10	1.1	50	5.4	927
Total	90	5.6	99	6.1	1,616

Grouped by characters of age. Group A: gestational age (GA): ≤32 weeks, Group B: 32 weeks < GA < 37 weeks, Group C: 37 weeks < GA < 42 weeks.

### 3.3 Univariate analysis of early wheezing in each group

#### 3.3.1 Premature group of GA ≤ 32 weeks

Since the birth weight of preterm infants with a gestational age of less than 32 weeks is usually less than 2,500 g. In this group, we used the relationship between BW and GA ([small-for-gestational-age (SGA), appropriate-for-gestational-age (AGA)] instead of BW for evaluation. The results showed the proportion of SGA infants in early wheezing group was significantly higher than that in normal group (*X^2^* = 8.154, *P* < 0.05) (Table 3).

**Table 3:** Univariate analysis for risk associated with early wheezing in preterm infants (GA ≤ 32 weeks).

Independent variable	Early wheezing (%)	No wheezing events (%)	*X* ^2^	*P*
Gender			0.169	>0.05
Male	20 (54.1)	58 (58.0)		
Female	17 (45.9)	42 (42.0)		
Mode of delivery			1.861	>0.05
Natural labor	17 (45.9)	59 (59.0)		
Cesarean section	20 (54.1)	41 (41.0)		
BW-GA			8.154	<0.05^a^
SGA	12 (32.4)	12 (12.0)		
AGA	25 (67.6)	88 (88.0)		
Breast feeding (<3 months)			0.078	>0.05
Yes	25 (67.6)	65 (65.0)		
No	12 (32.4)	35 (35.0)		
Personal history of allergy			2.897	>0.05
Yes	16 (43.2)	28 (28.0)		
No	21 (56.8)	72 (72.0)		
Family history of allergy			1.248	>0.05
Yes	8 (21.6)	16 (16.0)		
No	29 (78.4)	84 (84.0)		
Invasive mechanical ventilation			3.399	>0.05
Yes	25 (67.6)	50 (50.0)		
No	12 (32.4)	50 (50.0)		
Passive smoking			0.200	>0.05
Yes	21 (56.8)	61 (61.0)		
No	16(43.2)	39(39.0)		

BW, birth weight; GA, gestational age; BW-GA, the relationship between BW and GA; SGA, small-for-gestational-age; AGA, appropriate-for-gestational age.

^a^ indicate significant results with *P* value < 0.05.

#### 3.3.2 Premature group of 32 weeks < GA < 37 weeks

The proportion of male (*X*^2^ = 17.686, *P* < 0.05), the positive rate of personal history of allergy (*X*^2^ = 7.350, *P* < 0.05), the positive rate of family history of allergy (*X*^2^ = 12.797, *P* < 0.05) in the early wheezing group were significantly higher than those in normal group (Table 4).

**Table 4:** Univariate analysis for risk associated with early wheezing in preterm infants (32 weeks < GA < 37 weeks).

Independent variable	Early wheezing (%)	No wheezing events (%)	*X* ^2^	*P*
Gender			17.686	<0.05^a^
Male	70 (76.1)	242 (52.6)		
Female	22 (23.9)	218 (47.4)		
Mode of delivery			0.254	>0.05
Natural labor	35 (38.0)	188 (40.9)		
Cesarean section	57 (62.0)	272 (59.1)		
BW (<2.5 kg)			0.002	>0.05
Yes	56 (60.9)	279 (60.7)		
No	36 (39.1)	181 (39.3)		
Breast feeding (<3 months)			0.071	>0.05
Yes	46 (50.0)	237 (51.5)		
No	46 (50.0)	223 (48.5)		
Personal history of allergy			7.350	<0.05^a^
Yes	47 (51.1)	166 (36.1)		
No	45 (48.9)	294 (63.9)		
Family history of allergy			12.797	<0.05^a^
Yes	33 (35.9)	88 (19.1)		
No	59 (64.1)	372 (80.9)		
Invasive mechanical ventilation			0.443	>0.05
Yes	5 (5.4)	18 (3.9)		
No	87 (94.6)	442 (96.1)		
Passive smoking			0.000	>0.05
Yes	48 (52.2)	240 (52.2)		
No	44(47.8)	220(47.8)		

GA, gestational age; BW, birth weight.

^a^ indicate significant results with *P* value < 0.05.

#### 3.3.3 Full-term group of 37 weeks ≤ GA < 42 weeks

The proportion of male (*X*^2^ = 8.486, *P* < 0.05), the positive rate of personal history of allergy (*X^2^* = 3.949, *P* < 0.05), and the positive rate of family history of allergy (*X*^2^ = 6.126, *P* < 0.05) in the early wheezing group were significantly higher than those in normal group (Table 5).

**Table 5:** Univariate analysis for risk associated with early wheezing in full-term infants (37 weeks ≤ GA < 42 weeks).

Independent variable	Early wheezing (%)	No wheezing events (%)	*X* ^2^	*P*
Gender			8.486	<0.05^a^
Male	47 (78.3)	515 (59.4)		
Female	13 (21.7)	352 (40.6)		
Mode of delivery			0.123	>0.05
Natural labor	41 (68.3)	611 (70.5)		
Cesarean section	19 (31.7)	256 (29.5)		
BW (<2.5 kg)			0.304	>0.05
Yes	1 (1.7)	25 (2.9)		
No	59 (98.3)	842 (97.1)		
Breast feeding (<3 months)			1.046	>0.05
Yes	31 (51.7)	389 (44.9)		
No	29 (48.3)	478 (55.1)		
Personal history of allergy			3.949	<0.05^a^
Yes	23 (38.3)	230 (26.5)		
No	37 (61.7)	637 (73.5)		
Family history of allergy			6.126	<0.05^a^
Yes	18 (30.0)	150 (17.3)		
No	42 (70.0)	717 (82.7)		
Passive smoking			0.477	>0.05
Yes	29 (48.3)	459 (52.9)		
No	31 (51.7)	408 (47.1)		

GA, gestational age; BW, birth weight.

^a^ indicate significant results with *P* value < 0.05.

### 3.4 Multivariate analysis of early wheezing in each group

**Figure 2:**
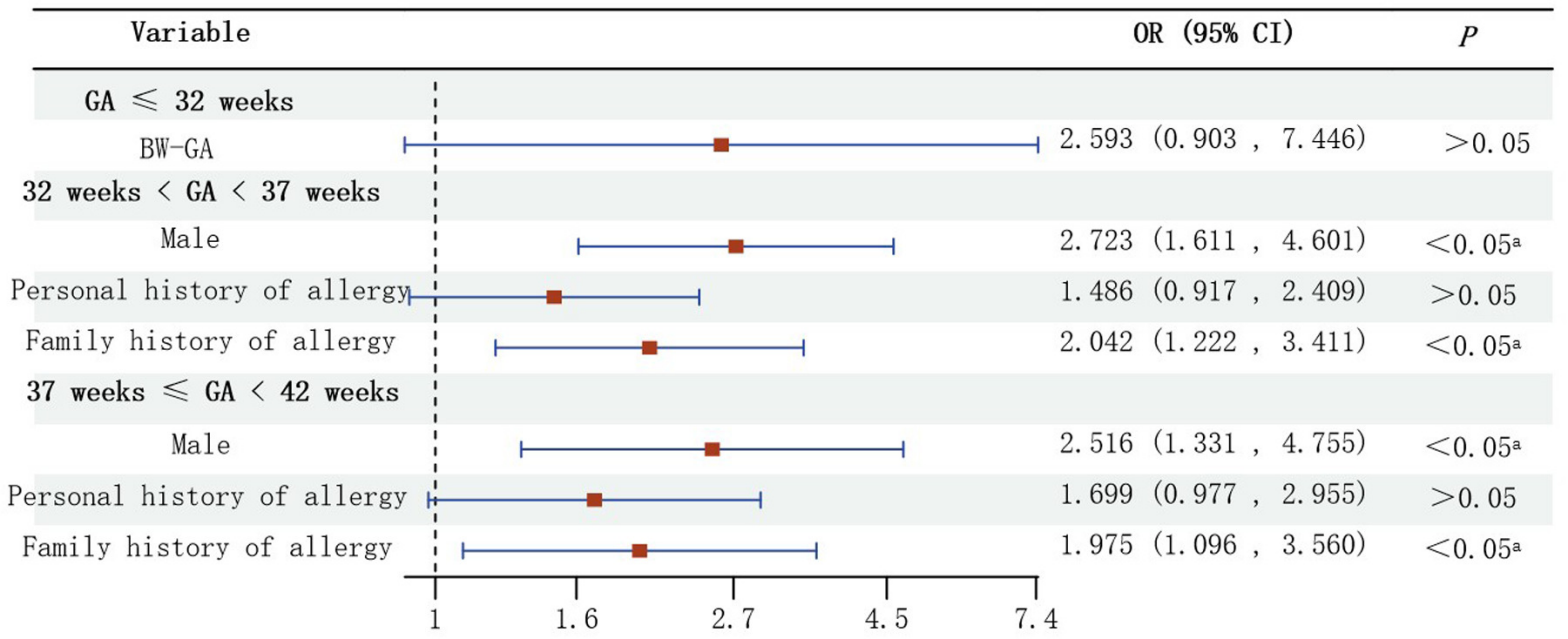
Multivariate analysis of early wheezing in infants (GA ≤ 32 weeks, 32 weeks < GA < 37 weeks and 37 weeks ≤ GA < 42 weeks).

Univariate analysis showed that the relationship between BW and GA was the possible factor influencing early wheezing in preterm infants with GA ≤ 32 weeks, but further analysis of its effect by the method of logistic regression showed no significant difference (Figure 2).

Male (*OR* = 2.723, *95% CI*: 1.611–4.601), family history of allergy (*OR* = 2.042, *95% CI*: 1.222–3.411) were risk factors for early wheezing in preterm infants with 32 weeks < GA < 37 weeks (Figure 2).

Male (*OR* = 2.516, *95% CI*: 1.331–4.755), family history of allergy (*OR* = 1.975, *95% CI*: 1.096–3.560) were risk factors for early wheezing in full-term infants with 37 weeks ≤ GA < 42 weeks (Figure 2).

## 4 Discussion

It has been confirmed that premature birth can cause immature lung, low lung function, immature immune system function, thereby increasing the risk of wheezing in children (6, 11–14). Enrico Lombard et al. showed that GA and the development of the lung were closely related (15). In this study, included newborns were grouped and compared by GA, and the results confirmed that newborns with younger GA were at higher risk of early wheezing, which was consistent with the study of Unal et al. (16). However, the incidence of early wheezing in different GA groups in this study was lower than that reported in the previous literature (17, 18), considering that it might be related to the short follow-up and the small sample size.

This study showed that the incidence of early transient wheezing in preterm infants with GA ≦ 32 weeks was significantly greater than that of preterm infants with 32 weeks < GA < 37 weeks. However, there was no significant difference in the incidence of early persistent wheezing between the two groups. The reason for this result may be that the respiratory system of preterm infants gradually develops with age, resulting in a decrease in early persistent wheezing (19). In addition, this study also found that the incidence of early persistent wheezing in preterm infants was significantly higher than that in full-term infants, which was consistent with previous literature reports (17). This study further demonstrated that preterm birth was a significant risk factor for wheezing episodes in children. While some preterm infants with GA ≦ 32 weeks exhibited transient wheezing that improved with pulmonary maturation, this preterm infants with GA ≦ 32 weeks cohort nevertheless maintains a higher risk of persistent wheezing compared to preterm infants with 32 weeks < GA < 37 weeks. Those findings confirmed an inverse relationship between gestational age and wheezing susceptibility, underscoring the need for enhanced respiratory monitoring throughout childhood in these high-risk populations. However, it is with great regret that we were unable to conduct a multivariate analysis due to the small sample size in each group.

Birth weight is a well-established indicator of prenatal growth, intrauterine nutritional status and maternal health. It is a sensitive indicator of fetal respiratory and immune system development (20). Global Initiative for Asthma (GINA) also added low birth weight as a risk factor for persistent airflow limitation (2). In this study, the mean birth weights were: 1,415.5 ± 170.0 g for preterm infants with GA ≤ 32 weeks, 2,395.1 ± 530.0 g for preterm infants with 32 weeks < GA < 37 weeks, and 3,210.2 ± 125.0 g for full-term infants, demonstrating the expected correlation between gestational age and birth weight. In the ≤32-week cohort, univariate analysis revealed SGA infants comprised 32.4% (12/37) of wheezing cases vs. 12.0% (12/100) in non-wheezing controls (*χ*^2^ = 8.154, *p* < *0.05*). However, binary logistic regression failed to maintain this significant association, potentially due to limited sample size. For preterm infants with 32 weeks < GA < 37 weeks and full-term infants, stratification by weight-for-gestational age was precluded by small subgroup numbers. Subsequent analysis using a 2.5 kg threshold showed no significant association with wheezing risk—a finding inconsistent with previous literature (21).

Some studies have shown that preterm infants with very low birth weight had a high incidence of impaired lung function, and the degree of impaired lung function was more severe in preterm infants with recurrent wheezing attacks (11, 22). However, because this study was a retrospective study, most of the included neonates lacked lung function data. The relationship between early wheezing and lung function in preterm infants is expected to be further demonstrated by a large sample of prospective studies in the future.

Gender was another risk factor for early wheezing in infants. In this study, we found that the proportion of males in the early wheezing group was significantly higher than that in normal group in both preterm infants with 32 weeks < GA < 37 weeks and full-term infants. Meanwhile, the results of univariate analysis and multivariate analysis showed that male was a possible influencing factor and risk factor for early wheezing, respectively. This was in line with previous studies (17, 23). However, this study did not find an association between gender and early wheezing in preterm infants with GA ≤ 32 weeks.

Personal history of allergy was a risk factor for wheezing in children (2). We also found that newborns with a personal history of allergy had a higher risk of early wheezing whether they were preterm infants with 32 weeks < GA < 37 weeks or full-term infants, which was consistent with previous studies (24). However, personal history of allergy did not show a significant difference in early wheezing in preterm infants with GA ≤ 32 weeks, suggesting that personal history of allergy might not be associated with early wheezing in preterm infants with GA ≤ 32 weeks. Family history of allergy was also an important risk factor for wheezing in children (25). The result of this study showed that family history of allergy was a risk factor for early wheezing in preterm infants with 32 weeks < GA < 37 weeks, which was consistent with previous findings (24). However, there was no correlation between family history of allergy and early wheezing of preterm infants with GA ≤ 32 weeks in this study, which was consistent with previous reports (17). This suggested that a family history of allergy might not be associated with early wheezing in preterm infants with GA ≤ 32 weeks. The above results indicated that the main cause of early wheezing in preterm infants with GA ≤ 32 weeks might be immature respiratory system, rather than personal history of allergy and family history of allergy.

In this study, these findings supported a gestational age-dependent dichotomy in wheezing pathogenesis. In preterm infants with GA ≤ 32 weeks, the pathogenesis of wheezing primarily stemmed from developmental lung pathology, whereas in preterm infants with 32 weeks < GA < 37 weeks, the underlying mechanisms transition towards conventional allergic pathways. This finding provides important guidance for clinical practice. For preterm infants with GA ≤ 32 weeks, focused monitoring of pulmonary development and growth trajectories may hold greater clinical relevance than routine allergy screening. In contrast, preterm infants with 32 weeks < GA < 37 weeks require preventive strategies aligned with those for term infants, including atopy risk assessment. Therefore, the wheezing pathogenesis will be further verified with a large sample size and explored mechanistic differences in the future.

This study found no statistically significant associations between caesarean delivery, tobacco exposure, artificial feeding, or invasive mechanical ventilation with wheezing incidence across gestational age subgroups. It may be attributable to diminished statistical power following gestational age stratification, Enhanced parental awareness of secondhand smoke risks, coupled with reduced tobacco exposure in children and advancements in modern infant formula composition, were likely contribute to a reduction in wheezing episodes.

This study has several limitations that warrant consideration. Firstly, this study indeed suffers from recall bias. Given that the history of wheezing in some children was investigated only after the age of 6, parents' memories of symptoms during infancy and early childhood may be incomplete, particularly for mild or early wheezing episodes. Additionally, differences in the timing of diagnosis, awareness of family history, and subsequent changes in health status may further affect the accuracy of parents' recall of related symptoms. To mitigate such biases, we implemented the following measures during the study: First, we cross-verified data with medical records to ensure the objectivity and reliability of the information. Second, we provided specific symptom examples (e.g., “wheezing sounds like a whistle”) to help parents recall and describe related symptoms more accurately. While we implemented standardized questionnaires and cross-verification with clinical records where possible, the inherent subjectivity of recalled data remains a constraint. Future prospective designs incorporating real-time digital symptom tracking (e.g., mobile health platforms) could mitigate this issue. Additional limitations include the need for a larger sample size across gestational age strata—particularly for preterm infants—as well as the influence of confounding variables. Despite these constraints, our findings provide meaningful insights into the risk factors for early wheezing in preterm infants. In the future, we anticipate better prospective cohort studies to mitigate recall bias.

## 5 Conclusion

Lower gestational age is associated with higher wheezing risk. Preterm infants have higher persistent wheezing incidence than full-term infants. Preterm infants with gestational age <32 weeks have higher transient wheezing incidence than those with gestational age 32–37 weeks or full-term infants. In preterm infants <32 weeks, small for gestational age (SGA) is a potential factor for wheezing. In preterm infants aged 32–37 weeks, male sex, personal allergy history, and family allergy history are potential factors for wheezing, with male sex and family allergy history being significant risk factors.

## Data Availability

The original contributions presented in the study are included in the article/Supplementary Material, further inquiries can be directed to the corresponding author.
