# Supplementary material for: Risk factors for early wheezing in preterm infants: a retrospective cohort study
Source: Front Pediatr. 2025 Jun 24;13:1555834. doi: 10.3389/fped.2025.1555834 (PMC12234546; doi:10.3389/fped.2025.1555834)
Supplement: Supplementary file 1 [file Supplementaryfile1.docx]

Appendix 1. Demographic Data Table

| Appendix 1. Demographic Data TableName | Date of Birth | Gestational Age (weeks) | Sex | Birth Weight (grams) | Mode of Delivery | Invasive Mechanical Ventilation | Follow-up Date | Feeding Method | Personal Allergy History | Parental Allergy History | Passive Smoking | Wheezing History |
| --- | --- | --- | --- | --- | --- | --- | --- | --- | --- | --- | --- | --- |
| Patient 1 |  |  |  |  |  |  |  |  |  |  |  |  |
| Patient 2 |  |  |  |  |  |  |  |  |  |  |  |  |
| Patient 3 |  |  |  |  |  |  |  |  |  |  |  |  |

Note：

1.Mode of Delivery: Categorized into vaginal delivery and cesarean section.

2.Feeding Method: Whether exclusively breastfed for <3 months.

3.Personal Allergy History: Includes eczema, allergic rhinitis, food allergy, allergic asthma, atopic dermatitis, etc.

4. Parental Allergy History: First-degree relatives with asthma or allergic rhinitis.

5. Passive Smoking: Presence of smokers among cohabitants.

6. Wheezing: Includes frequency of wheezing, age at first and last wheezing episode, and diagnosis at the time.

Appendix 2. Pairwise comparison (Bonferroni) of the incidence of early wheezing in each group.

| **Groups** | ***X*^2^** | ***P*** |
| --- | --- | --- |
| Group A vs Group B | 7.713 | < 0.0167^a^ |
| Group A vs Group C | 60.747 | < 0.0167^a^ |
| Group B vs Group C | 38.992 | < 0.0167^a^ |

“a” indicate significant results with *P* value < 0.0167.

The significant level of the incidence of early wheezing in the table is 0.0167 (0.05/3).

Grouped by characters of age. Group A: gestational age (GA): ≤ 32 weeks, Group B: 32 weeks < GA < 37 weeks, Group C: 37 weeks < GA < 42 weeks.

Appendix 3. Pairwise comparison (*Bonferroni*) of the incidence of persistent early wheezing in each group.

| **Groups** | ***X^2^*** | ***P*** |
| --- | --- | --- |
| Group A vs Group B | 0.389 | > 0.0167 |
| Group A vs Group C | - | < 0.0167^a^ |
| Group B vs Group C | 77.012 | < 0.0167^a^ |

“-” indicate that it is not suitable for chi-square test, but Fisher's exact test.

“a” indicate significant results with *P* value < 0.0167.

The significant level of the incidence of early persistent wheezing in the table is 0.0167 (0.05/3).

Grouped by characters of age. Group A: gestational age (GA): ≤ 32 weeks, Group B: 32 weeks < GA < 37 weeks, Group C: 37 weeks < GA < 42 weeks.

Appendix 4. Pairwise comparison (*Bonferroni*) of the incidence of transient early wheezing in each group.

| **Groups** | ***X*^2^** | ***P*** |
| --- | --- | --- |
| Group A vs Group B | 11.818 | < 0.0167^a^ |
| Group A vs Group C | 14.136 | < 0.0167^a^ |
| Group B vs Group C | 0.001 | > 0.0167 |

“a” indicate significant results with *P* value < 0.0167.

The significant level of the incidence of transient early wheezing in the table is 0.0167 (0.05/3).

Grouped by characters of age. Group A: gestational age (GA): ≤ 32 weeks, Group B: 32 weeks < GA < 37 weeks, Group C: 37 weeks < GA < 42 weeks.
